# Importance of lymph node ratio in HPV-related oropharyngeal cancer patients treated with surgery and adjuvant treatment

**DOI:** 10.1371/journal.pone.0273059

**Published:** 2022-08-12

**Authors:** Kyu Hye Choi, Jin Ho Song, Ji Hyun Hong, Youn Soo Lee, Jin Hyoung Kang, Dong-Il Sun, Min-Sik Kim, Yeon-Sil Kim

**Affiliations:** 1 Department of Radiation Oncology, Seoul St. Mary’s Hospital, College of Medicine, The Catholic University of Korea, Seoul, Korea; 2 Department of Pathology, Seoul St. Mary’s Hospital, College of Medicine, The Catholic University of Korea, Seoul, Korea; 3 Department of Medical Oncology, Seoul St. Mary’s Hospital, College of Medicine, The Catholic University of Korea, Seoul, Korea; 4 Department of Otolaryngology–Head and Neck Surgery, Seoul St. Mary’s Hospital, College of Medicine, The Catholic University of Korea, Seoul, Korea; MD Anderson Cancer Center, UNITED STATES

## Abstract

**Objectives:**

The pathologic nodal stage of human papillomavirus (HPV)-related oropharyngeal cancer (OPC) patients is classified according to the number of lymph nodes (LNs), as revised in 2018. Previous studies showed that the LN ratio (LNR) could be also a significant prognostic factor in head and neck cancer, but there are few studies on the LNR in HPV-related [HPV(+)] OPC. The aim of the present study was to analyze the predictive value of the LNR for survival and recurrence in HPV(+) OPC patients.

**Materials and methods:**

HPV(+) OPC patients treated with surgery with or without postoperative radiotherapy from January 2000 to March 2019 were evaluated. The patients were divided into two sets of three groups, according to LN numbers based on pathologic nodal stages, and LNRs by a cutoff value of 0.05. The medical records were reviewed, and the overall survival (OS), disease-free survival, locoregional recurrence, and distant metastasis incidence were analyzed.

**Results:**

Ninty patients were included and the median follow-up period was 38.2 months. There were no significant differences in OS in the LN number groups. However, there was a significant difference in OS in the LNR groups (P = 0.010). The incidence of distant metastasis in the LNR groups was significantly different (P = 0.005).

**Conclusion:**

The LNR in HPV(+) OPC patients may be a more useful tool to predict survival and distant metastasis than the LN number. Additional research and consensus on surgical pathology are needed before applying the LNR to adjuvant treatment decisions and pathologic nodal staging.

## Introduction

The incidence of human papillomavirus (HPV)-related oropharyngeal cancer (OPC) increases 5% annually worldwide, and more outbreaks of HPV-related head and neck cancer are predicted in the near future in developed countries [1, 2]. HPV infects the basal keratinocytes of the oropharyngeal mucous membranes, expressing E6 and E7 oncoproteins causing carcinogenesis. HPV-related [HPV(+)] OPC shows basal squamous cell carcinoma in the histopathological findings and is known to occur in younger people with less smoking exposure. The usual clinical features of HPV(+) OPC manifest as early-stage primary tumors and relatively advanced LN metastasis.

The overall survival and disease-free survival of HPV(+) OPC patients were superior to HPV-negative [HPV(–)] OPC patients in several prospective studies [3, 4]. A meta-analysis demonstrated that HPV infection was a good independent prognostic factor of oropharyngeal cancer [5, 6]. This was introduced as a new stage in the eighth edition of the American Joint Committee on Cancer (AJCC), separate from HPV(–) OPC [7]. Nodal staging in the seventh AJCC edition is classified by laterality and size, but the number of metastatic lymph nodes (LNs) is used in the recent eighth edition. In addition, the pathological stages of the nodes are considered separately from clinical nodal stage [8, 9].

The number of metastatic lymph nodes can be influenced by how extensively the cervical lymph nodes are harvested surgically. If fewer cervical lymph nodes are resected, the pathologic stage may be underestimated. AJCC staging suggests that more than 10 LNs should be removed in selective neck dissections (SNDs) and 18 or more in radical neck dissections (RNDs) in HPV(+) OPC patients as appropriate neck dissection. Zenga et al. [10] showed that at least 26 nodes harvested during neck dissection may improve the risk assessment of regional disease.

The LN ratio (LNR) presents the ratio of metastatic LNs among the LN yield. This means that the LN metastasis burden, rather than the LN number, and is expected to be a predictor of prognosis in patients with head and neck cancer [11]. However, there are few studies on the predictive power of the LNR in HPV(+) OPC patients. As the number of metastatic nodes in HPV(+) OPC affects the prognosis more than size, the predictive power of the LNR in HPV(+) OPC is assumed to be higher than that in other head and neck cancers because of relatively advanced LN metastasis [8]. The aim of this study was to validate the eighth AJCC pathologic nodal staging system and to compare the prognosis of HPV(+) OPC by LNR and LN number.

## Materials and methods

This was a retrospective study that reviewed the surgical pathology of HPV(+) OPC patients operated on by two head and neck surgeons with over 20 years of surgical experience at a single institution. The inclusion criteria were: 1) pathologically confirmed HPV(+) oropharyngeal squamous cell carcinoma, 2) primary tumor stage T1-4 without distant organ metastasis at the pretreatment diagnosis, 3) patients who underwent wide excision and LN dissection followed by adjuvant treatment as needed, and 4) patients aged 18–75 years with Eastern Cooperative Oncology Group performance status ≤ 2. The patients who received induction chemotherapy before surgery or incomplete surgery were excluded from this study.

Among the 252 OPC patients who underwent surgery at Seoul St. Mary’s Hospital between January 2000 and March 2019, 90 patients with HPV(+) OPC were included in the analysis except 36 patients who underwent neoadjuvant chemotherapy and 126 patients with HPV(–) or unknown HPV status. The HPV status of all 90 HPV(+) patients was confirmed through p16 immunohistochemistry, of which 79 were HPV positive either through *in situ* hybridization or polymerase chain reaction additionally. The numbers of dissected and metastatic lymph nodes obtained as surgical specimens were reviewed. SND was performed in the clinically negative node patients and RND or modified RND (MRND) was performed in the clinically positive node patients. Cervical lymphadenectomy was performed bilaterally or ipsilaterally with more than 15 lymph nodes removed by RND or MRND and more than 10 lymph nodes from SND. A median of 38 RND or MRND nodes and 23 SND nodes were harvested in this study. The AJCC staging system eighth edition was used in this study.

Adjuvant treatment was considered if the primary tumor was T3/T4 stage, the resection margin was insufficient (< 5 mm), extracapsular invasion or multiple nodal metastases were found. Radiation was delivered using intensity modulated radiation therapy with a simultaneous integrated boost. A total of 60 Gy was prescribed for high-risk lesions in the primary tumor bed or positive nodal area, and 54 to 58 Gy in the low-risk area of the neck. Patients with positive resection margin or extranodal extension (ENE) were treated with up to 63–66 Gy.

Patient medical records were reviewed for the recurrence and survival of the patients during the follow-up period. Overall survival (OS) was defined as the interval from the time of surgery to death or the last follow-up, and disease-free survival (DFS) was defined as the interval between the time of surgery and relapse or the last follow-up. The recurrence of the oropharyngeal region or regional lymph node involvement was classified locoregional recurrence (LRR), and the incidence of metastasis to distant organs or non-regional lymph nodes was classified distant metastasis (DM). The patients visited the physicians every one to three months for the first year, then every three to six months until three years, and then annually for physical examination, CT or MRI imaging, or biopsy for suspected recurrence.

The ratio of resected lymph nodes to metastatic lymph nodes was defined as the LNR, and OS and DFS were compared between the three LNR groups. In addition, survival analysis was performed by dividing the three groups according to the pathological eighth edition of AJCC lymph node stages. We also compared the incidence of LRR and DM in each group. Kaplan-Meier curves were obtained from survival analysis and analyzed by the log-rank test. Cox regression analysis was performed for factors affecting OS. Multivariate analysis was performed using the Cox Proportional hazards model for prognostic factors that had a *p*-value less than 0.1 in the univariate analysis. All statistical tests with p-values less than 0.05 were considered statistically significant. SPSS version 24 (IBM, Armonk, NY) was used for statistical analyses. This study was approved by the Institutional Review Board (IRB) of Seoul St. Mary’s Hospital (IRB No. KC20RISI0005). The requirement for informed consent was waived by the IRB due to the retrospective nature of the study.

## Results

### Patients and tumor characteristics

A total of 90 patients were included for analysis in this study for a median follow-up period of 38.2 months (range, 3–234 months). T1-2 tumors were found in 79 patients (87.7%) and regional lymph node metastasis was found in 77 patients (85.5%). The median number of harvested lymph nodes was 50 nodes (range, 15–124), and the median number of metastatic lymph nodes was two nodes (range, 1–43). The characteristics of the included patients are summarized in **Table 1**.

**Table 1:** Patient, tumor, and treatment characteristics.

Characteristic			N (%)
Patient characteristics			
	Age		
		< 60	53 (58.9)
		≥ 60	37 (41.1)
	Sex		
		Male	73 (81.1)
		Female	17 (18.9)
	Smoking		
		No	39 (43.3)
		< 20 PPY	15 (16.7)
		≥ 20 PPY	36 (40.0)
Tumor characteristics			
	Primary tumor location		
		Tonsil	75 (83.3)
		BOT	13 (14.4)
		PPW	2 (2.2)
	pT stage (AJCC 8^th^)		
		T1	39 (43.3)
		T2	40 (44.4)
		T3	7 (7.8)
		T4	4 (4.4)
	Depth of invasion (mm)		
		≤ 10 mm	35 (38.9)
		> 10 mm	51 (56.7)
		N/A	4 (4.4)
	Resection margin		
		Negative	57 (63.3)
		Positive	25 (27.8)
		Close (< 5mm)	6 (6.7)
		N/A	2 (2.2)
	Lymphatic invasion		
		Negative	43 (47.8)
		Positive	45 (50.0)
		N/A	2 (2.2)
	Vascular invasion		
		Negative	81 (90.0)
		Positive	7 (7.8)
		N/A	2 (2.2)
	Perineural invasion		
		Negative	79 (87.8)
		Positive	9 (10.0)
		N/A	2 (2.2)
	pN stage (AJCC 8^th^)		
		N0	13 (14.4)
		N1	56 (62.2)
		N2	21 (23.3)
	pN size in N-positive		
		≤ 3 cm	41 (45.6)
		> 3 cm ≤ 6 cm	27 (30.0)
		> 6 cm	0
		N/A	9 (10.0)
		No lymph node involvement	13 (14.4)
	Extranodal extension		
		Negative	38 (42.2)
		Positive	36 (40.0)
		N/A	3 (3.3)
		No lymph node involvement	13 (14.4)
	Low neck involvement		
		Negative	68 (75.6)
		Positive	9 (10.0)
		No lymph node involvement	13 (14.4)
Treatment characteristics			
	Primary op method		
		WE	48 (53.3)
		TORS	42 (46.7)
	Ipsilateral neck op method		
		MRND	72 (80.0)
		SND	16 (17.8)
		RND	2 (2.2)
	Contralateral neck op method		
		Not-done	29 (32.2)
		MRND	8 (8.9)
		SND	53 (58.9)
	Adjuvant treatment		
		No RT	17 (18.9)
		RT alone	26 (28.9)
		CCRT	47 (52.2)

PPY, per-pack-year; BOT, base of tongue; PPW, posterior pharyngeal wall; AJCC, American Joint Committee on Cancer; N/A, not available; WE, wide excision; TORS, transoral robotic surgery; MRND, modified radical neck dissection; SND, selective neck dissection; RND, radical neck dissection; RT, radiotherapy; CCRT, concurrent chemoradiotherapy.

### Determination of LNR

**Fig 1:**
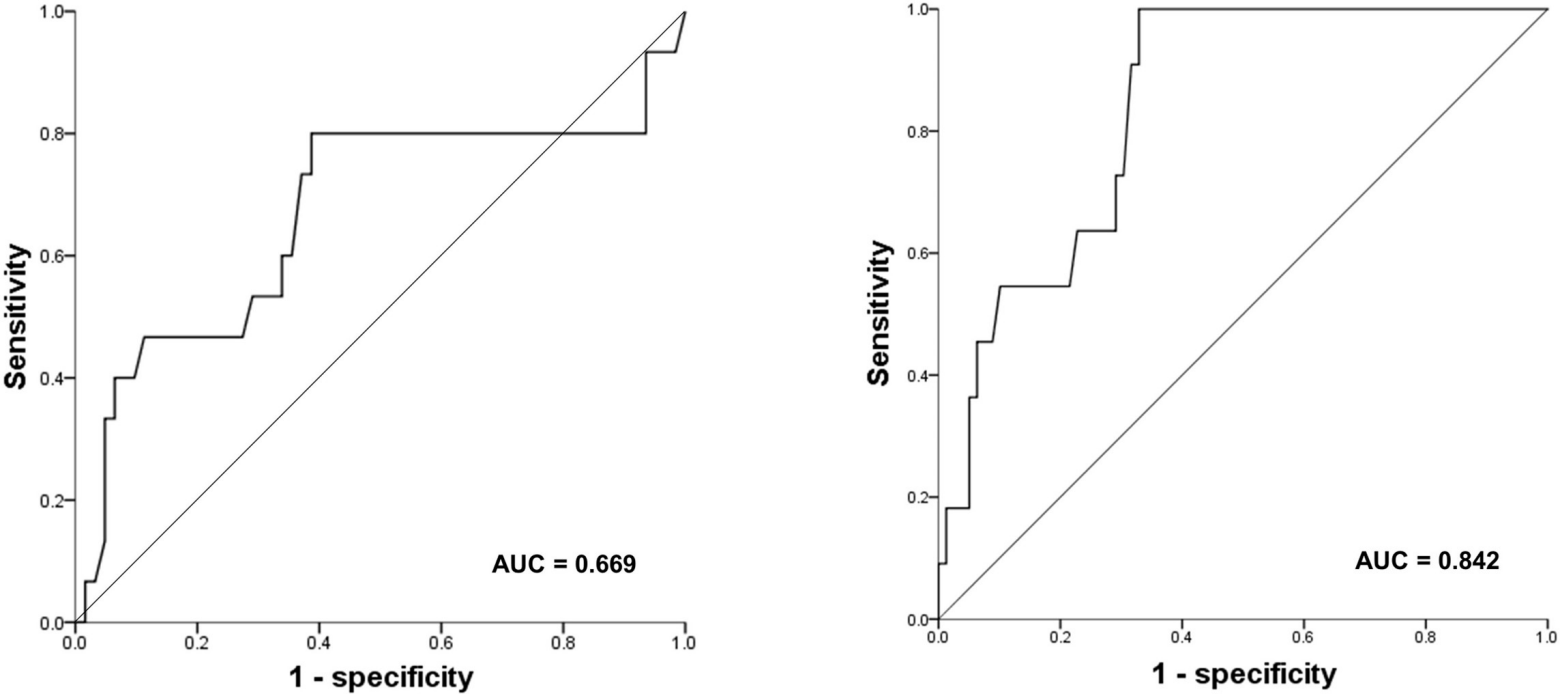
Receiver-operating characteristic curve analysis for the lymph node ratio predicting survival (A) and recurrence (B).

The median LNR, calculated as the ratio of metastatic lymph nodes to harvested lymph nodes, was 0.049 (range, 0.009–0.815) and 13 patients had no lymph node metastasis. Receiver operating characteristics (ROC) curves were generated to analyze the predictive power of the LNR for survival and recurrence. Areas under the curve (AUC) were 0.669 and 0.842, respectively (**Fig 1**). Survival analysis was performed according to the three LNR groups (LNR 0, LNR ≤ 0.05, LNR > 0.05) by setting a cutoff value of 0.05 with high sensitivity and specificity (sensitivity = 80.0%, specificity = 59.7% for survival, sensitivity = 86.7%, specificity = 66.7% for recurrence).

### Recurrence and survival analysis

There were 15 deaths and 11 recurrences in a total of 90 patients during the follow-up period. The time to relapse was a median of 15.4 months (range, 2.6–70.2), and the median time to death was 33.6 months (range, 4.7–76.9). The recurrence patterns showed five patients with LRR, 10 with DM, and 4 with LRR and DM at the same time.

**Fig 2:**
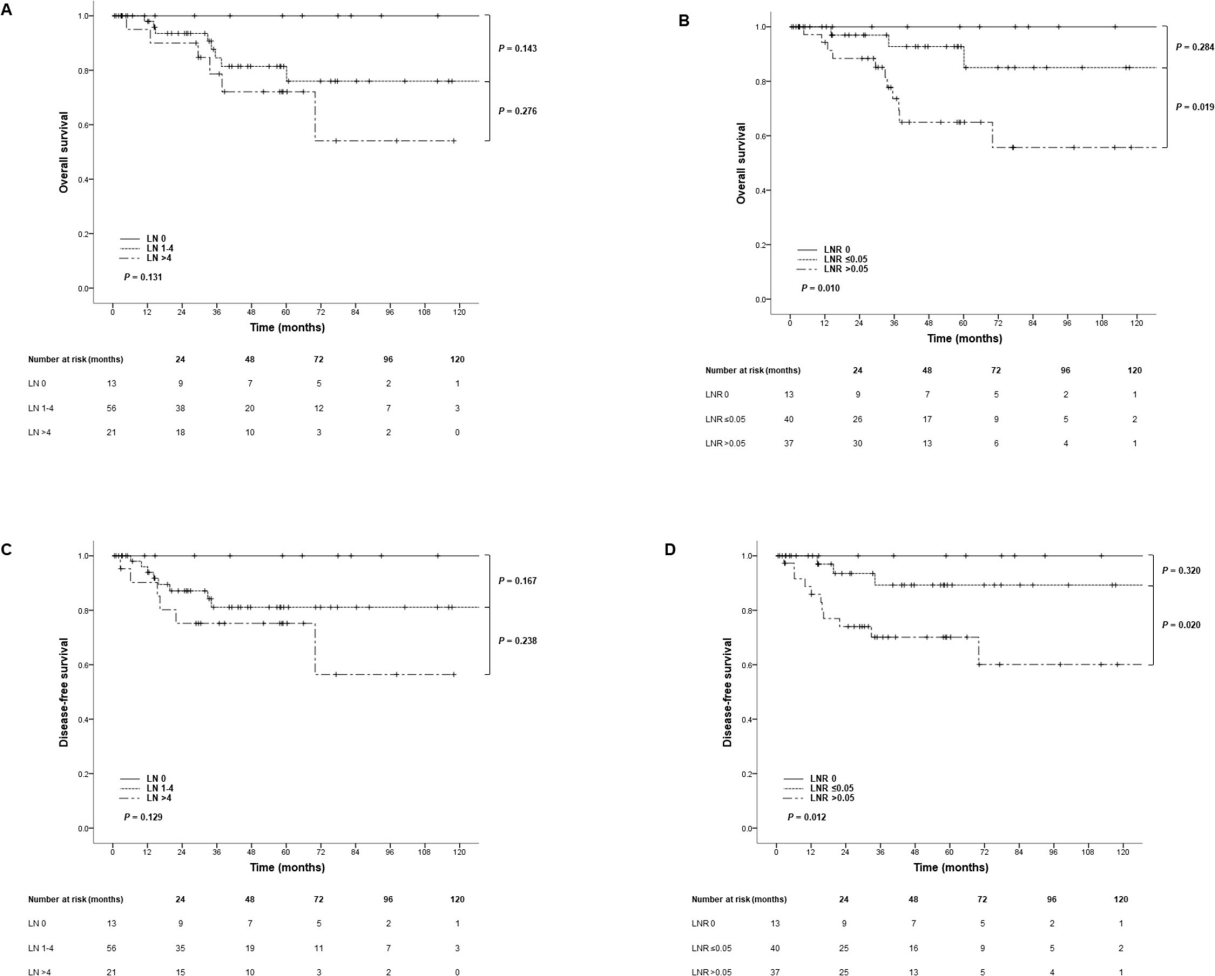
Kaplan-Meier curves of overall survival (OS) (A, B) and disease-free survival (DFS) (C, D) according to lymph node numbers and ratios.

Survival analysis was performed according to the number of LNs described by pathological lymph node staging in the AJCC eighth edition (LN 0, LN 1–4, LN > 4). The mean survival durations according to the three LN number groups were 59.2, 48.0, and 47.7 months, respectively. The five-year OS and DFS were 100% vs. 81.4% vs. 72.1% and 100% vs. 81.1% vs. 75.2%, respectively, in the three groups. There were no statistically significant difference between the three LN number groups in OS or DFS (*P* = 0.131 and *P* = 0.129). However, survival analysis of the three LNR groups (LNR 0, LNR ≤ 0.05, LNR > 0.05), showed significant differences in both OS and DFS (OS, *P* = 0.010; DFS, *P* = 0.012). **Fig 2** shows the Kaplan-Meier curves for OS and DFS in the LN number and ratio groups. The five-year OS and DFS rates on the LNR groups were 100% vs. 92.8% vs. 65.0% and 100% vs. 89.3% vs. 70.1%, in the LNR 0, LNR ≤ 0.05, LNR > 0.05 groups, respectively.

**Fig 3:**
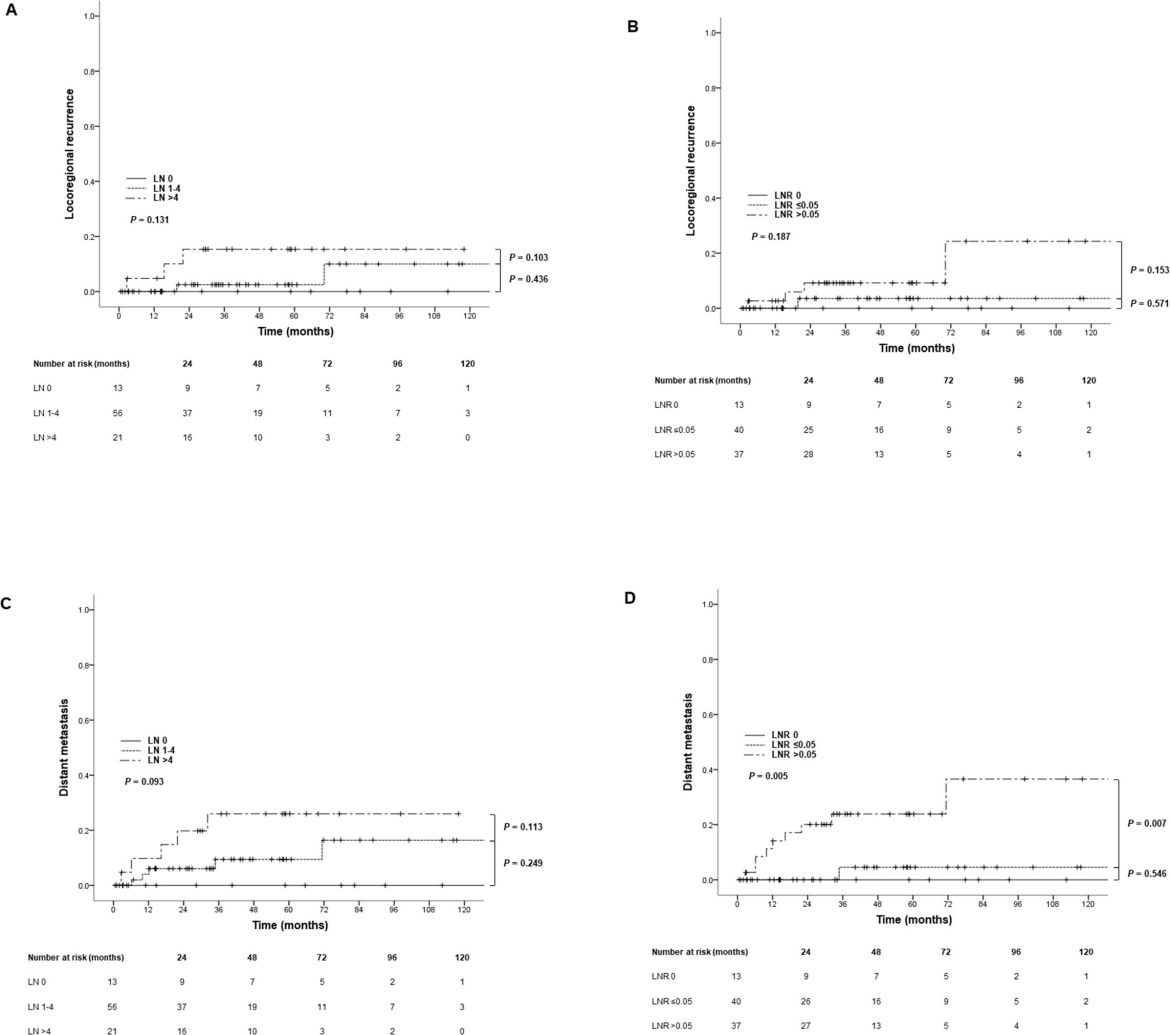
Kaplan-Meier curves of locoregional recurrence (LRR) (A, B) and distant metastasis (DM) (C, D) according to lymph node numbers and ratios.

Log-rank tests for the incidence of LRR and DM were performed to analyze the differences between the groups according to the recurrence patterns. The analysis of LRR did not show significant differences between the groups according to the number and LNRs (*P* = 0.131 in LN number, 0.187 in the LNR groups). The five-year DM incidence rates according to LN number (LN 0, LN 1–4, LN > 4) were 0%, 9.4%, and 26.0%, and those according to LNR (LNR 0, LNR ≤ 0.05, LNR > 0.05) were 0%, 4.5%, and 23.9%, respectively. Although there was a difference in DM rate, there were no statistically significant (*P* = 0.093). However, the results showed statistically significant differences according to the LNRs (*P* = 0.005). These findings are shown in **Fig 3**.

### Prognostic factors for overall survival

Additional Cox regression analyses were performed to analyze the factors related to survival (**Table 2**). In univariate analysis, surgical resection margin, perineural invasion, extranodal extension, and LNR were identified as significant prognostic factors. In multivariate analysis, resection margin and extranodal extension showed significant differences with hazard ratios of 5.942 (95% confidence interval, 1.286–27.453) and 7.014 (0.882–55.769), respectively.

**Table 2:** Cox-regression analysis predicting survival in HPV-related OPC.

Characteristic	Univariate	Multivariate analysis		
	*P-*value	*P*-value	HR	95% CI
Age, year old (≤ 60 vs. > 60)	0.562			
Smoking, pack-per-year (< 20 vs. ≥ 20)	0.214			
Primary surgery (wide excision vs. transoral robotic)	0.249			
Pathologic node metastasis (negative vs. positive)	0.054			
Pathologic node laterality (ipsilateral vs. both)	0.230			
Pathologic node multiplicity (1–4 vs. > 4)	0.674			
Pathologic T stage (pT1-2 vs. T3-4)	0.107			
Extranodal extension (negative vs. positive)	0.019	0.018	7.014	0.882–55.769
Pathologic node largest size, cm (≤ 6 vs. > 6)	0.620			
Pathologic node number (≤ 4 vs. > 4)	0.353			
Lymph node ratio (≤ 0.05 vs. > 0.05)	0.003	0.434		
Tumor grade (well or moderate vs. poor)	0.813			
Resection margin (negative vs. positive/close)	0.001	0.007	5.942	1.286–27.453
Lymphatic invasion (negative vs. positive)	0.082			
Vascular invasion (negative vs. positive)	0.803			
Perineural invasion (negative vs. positive)	0.029	0.322		
Adjuvant treatment (RT alone vs. CCRT)	0.221			
Ipsilateral neck dissection (vs. SND)	0.299			
Contralateral neck dissection (vs. SND)	0.753			

HR, hazard ratio; CI, confidence interval; RT, radiotherapy; CCRT, concurrent chemoradiotherapy; RND, radical neck dissection; MRND, modified RND; SND, selective neck dissection.

## Discussion

There has been a change in the treatment paradigm for HPV(+) OPC. Previous studies reported that HPV(+) OPC had a better prognosis and response to chemotherapy or radiotherapy than HPV(–) OPC [12–14]. Based on this, several randomized studies omitting adjuvant treatment or reducing the radiotherapy dose or field in HPV(+) OPC patients are underway, and the OPC treatment policy may change in the future depending on these results [15, 16]. The metastatic regional nodes in HPV(+) OPC are relatively cystic and bulky, and there are more positive lymph nodes but the prognosis is better than that for HPV(–) OPC patients [17–20]. Therefore, nodal metastasis in HPV(+) OPC is expected to have a different prognostic value than in HPV(–) OPC.

This study was designed to validate the newly revised eighth AJCC pathological lymph node staging system and assess the value of LNR in predicting survival and recurrence in HPV(+) OPC patients. Previous studies validating the eighth AJCC staging have been mainly based on definitive concurrent chemoradiation for AJCC staging, which lacks validation for pathological node staging through surgery [21–23]. Recently, Hobelmann et al. [24] conducted a validation of pathologic nodal staging in surgically-managed HPV(+) OPC by the eighth AJCC staging system. They reported that the pathologic nodal staging in the eighth AJCC edition was not associated with recurrence, but other LN parameters, such as extranodal extension, had more predictive power. Haughey et al. [9] analyzed the pathologic nodal staging of 704 patients in a multicenter study. There was no significant difference in survival based on pathologic node classification. We also analyzed the efficacy of the pathologic nodal stages in the eighth AJCC system, which are based on the number of involved LNs. Our results also could not confirm the prognostic power of LN numbers for OS and DFS. In this study, the number of patients was small compared to the previous multicenter studies, but relatively consistent pathological specimens by experienced surgeons were analyzed.

Several studies demonstrated the prognostic impact of LN in HPV(+) OPC [22, 25–27]. In previous studies, LN numbers, rather than the lymph node staging based on the seventh AJCC staging system, showed significant differences in survival rates [8, 9, 22]. Sinha et al. [8] reported that the number of metastatic nodes in pathologic specimens was an independent predictor of disease-specific survival, and the authors’ validation study by the International Collaboration on Oropharyngeal cancer Network for Staging (ICON-S) supported the finding that higher LN numbers could increase the risk of recurrence. However, they also discussed the importance of an accurate enumeration of pathologic metastatic nodes in surgically dissected positive nodes.

In the current study, among the factors for LN burden, the importance of the LNR as well as validation of the pN stages of resected HPV(+) OPC patients were evaluated. The AUC value for survival showed moderate significance in the ROC curve, however the value for recurrence showed relatively high accuracy. The LNR showed a significant difference of recurrence despite the small number of cases, indicating that the influence is expected to be greater in outcome predictions. In a previous multivariate analysis [28], the LNR of HPV(+) OPC did not predict OS or recurrence-free survival compared to HPV(–) OPC. They analyzed more patients with a locally advanced stage above T3 (28%) than in our study (12%), which is expected to have a greater impact on survival at T stage than LN burden. The present study aimed to analyze the effect of LN metastasis more intensively in HPV(+) OPC patients with relatively low T stage.

In the analysis of the recurrence patterns, high LNRs in HPV (+) OPC were identified to increase the risk of distant metastasis. Improvements in locoregional recurrence and survival have been reported in HPV (+) OPC compared to HPV (–) OPC patients, but not for distant metastasis in previous studies [3, 29, 30]. Chung et al. [31] performed Cox regression analysis to analyze the factors affecting DM in OPC patients and showed that the risk of DM was high in cases of low neck involvement and initial tumor sizes ≥ 3 cm. Reinisch et al. [32] reported that classifying patients for LNR with a cutoff of 0.06 resulted in significant differences in survival, and this effect of LNR was independently found in patients with extracapsular spread. This previous study on the effect of the LNR on survival did not report the effect on DM [32, 33]. In our study, the LNR was analyzed to determine the effect on distant metastasis in addition to survival.

Recently, some studies have reported the importance of ENE not included in the eighth AJCC lymph node staging [26, 27]. Miccio et al. [27] performed a Cox regression analysis for predicting the overall survival of resected HPV(+) OPC patients in a U.S. population. The nodal stage of the eighth AJCC edition, LN size, and ENE were significant prognostic factors, whereas node laterality did not affect the overall survival. Similarly, ENE was considered to be a significant factor in the Cox regression analysis of survival in the current study. Further studies on the predictive value of characteristics of LN metastasis such as large, bulky and matted LN, and the validation of pathologic nodal staging are needed.

The LNR in dissected nodes represents the extent of lymphatic spread and cervical nodal clearance. Lymphoangiogenesis is induced intratumorally by endothelial markers and results in regional metastasis in head and neck cancer [34, 35]. Sufficient nodal dissection may be used to inhibit lymphangiogenesis and the proportion of metastasized lymph nodes is expected to reflect the risk of regional metastasis [32]. However, there can be a limitation that the number of neck dissections varies from institution to institution or surgeon to surgeon, so the LNR is a relative concept rather than having absolute value. Jacobi et al. [33] suggested a new LNR parameter that complemented the LN yield (LNY). If the LNR^LNY^ is used as they suggested, the number of resected LNs can overcome the relative meaning of the LNR, but no further studies have been conducted. The study reported an improvement in OS in LNR ≤ 0.1 in HPV (+) OPC patients, whereas our study showed a difference in DM as well as OS based on a cutoff of 0.05. Our study had a higher number of dissected LNs compared to other studies, and showed high specficity and sensitivity at a lower cutoff value (0.05) than other studies (0.06–0.1). The significant differences of OS and DFS were also found when analyzed based on other cutoff values such as 0.06 or 0.1, but the difference in survival rate and low p-value was greatest when 0.05 was set as a cutoff value. However, no other cohorts have been evaluated. In addition, as a limitation of a retrospective study, variations in the resection numbers and selection bias cannot be excluded.

In this study, patients with node-positive and high T-stage underwent MRND or RND as a method of LN dissection, and large number of lymph node resections were performed. And also, due to the small number of events, the statistical superiority of LNR could not be proven in multivariate analysis. ENE and resection margin were found to be significant factors in the multivariate analysis of survival, and these two factors are considered major indications for adjuvant concurrent chemoradiotherapy (CCRT) in the current guidelines. In the Kaplan-Meier curve, LNR exhibited a significant difference in OS and RFS, and it is likely to be significant in multivariate analysis if a greater number of event subjects are evaluated.

In general, LNR could be considered more of a qualified metrics for assessing LN burden. However, it is highly dependent on surgeon and pathologist factors. Currently, it is difficult to consider as a practical staging system. There are varying cutoffs shown by multiple prior studies have looked at the improved accuracy of LNR. In the current study, the enrolled patients underwent surgery at one institution by only two surgeons to minimize dissection variation. Further studies on variables representing LN burden and dissection yield are needed.

## Conclusion

In conclusion, high LNR indicated high DM and low OS rates in HPV (+) OPC patients, which showed significant differences compared to LN number. It may play a significant role in pathologic nodal staging in HPV(+) OPC patients combined with LN number, although additional long-term follow-up and a prospective study are warranted. Further research and consensus on surgical pathology will be needed to apply the LNR to clinical treatment and pathologic staging.
